# Supplementary material for: Collagen constitutes about 12% in females and 17% in males of the total protein in mice
Source: Sci Rep. 2023 Mar 18;13:4490. doi: 10.1038/s41598-023-31566-z (PMC10024738; doi:10.1038/s41598-023-31566-z)
Supplement: Supplementary file 2 — Supplementary Table 2. [file 41598_2023_31566_MOESM2_ESM.docx]

Supplementary Table 2. Collagen content tissues relative to dry weight

| **Statement of % collagen of dry weight tissue** | **No citation or cited review article** | **References** |
| --- | --- | --- |
| Collagen accounts for 90% of scleral dry weight | x | [^1–10^](https://app.readcube.com/library/2cbcb46e-481b-4bc7-a6db-4d4d307cdf46/all?uuid=283696033089588&item_ids=2cbcb46e-481b-4bc7-a6db-4d4d307cdf46:f74701f5-d5f9-473a-ab21-703a94398fed,2cbcb46e-481b-4bc7-a6db-4d4d307cdf46:41fbc3a0-00d3-4d5c-bcef-15dec377e03c,2cbcb46e-481b-4bc7-a6db-4d4d307cdf46:f14043f5-0cd1-43f9-9f4b-10b97361906e,2cbcb46e-481b-4bc7-a6db-4d4d307cdf46:2ed9e20e-177b-4978-b035-d13e175f0a82,2cbcb46e-481b-4bc7-a6db-4d4d307cdf46:97f7de6a-cf98-4787-94be-3bf77d7107cb,2cbcb46e-481b-4bc7-a6db-4d4d307cdf46:b2189e6b-07ef-42d0-abd4-b6f3ed6d1e11,2cbcb46e-481b-4bc7-a6db-4d4d307cdf46:cbee768c-616b-4bc6-8ac9-a1f065ad2a51,2cbcb46e-481b-4bc7-a6db-4d4d307cdf46:4dc2fe06-a2f0-4e92-92cd-a135a268042f,2cbcb46e-481b-4bc7-a6db-4d4d307cdf46:6b2be576-63b9-40b7-a40b-ddf2e46d2cb3,2cbcb46e-481b-4bc7-a6db-4d4d307cdf46:7ac2f813-7ae5-4002-9d5d-cfe27967fb2c) |
| Of the tendon dry weight, 60–85% is collagen | x | [^11–23^](https://app.readcube.com/library/2cbcb46e-481b-4bc7-a6db-4d4d307cdf46/all?uuid=3807277979765483&item_ids=2cbcb46e-481b-4bc7-a6db-4d4d307cdf46:a1b78eb5-d097-4ca6-a4d8-e3b794e81b1f,2cbcb46e-481b-4bc7-a6db-4d4d307cdf46:f02b79f7-19e3-41b4-a589-d58c4e0e8d27,2cbcb46e-481b-4bc7-a6db-4d4d307cdf46:8cdbedf6-e4b3-449b-af44-721a6874026f,2cbcb46e-481b-4bc7-a6db-4d4d307cdf46:78ae0984-257b-4b3e-9f37-9fe330eada0c,2cbcb46e-481b-4bc7-a6db-4d4d307cdf46:5e635cc6-b660-4aa5-ab2b-bdbbdf653d70,2cbcb46e-481b-4bc7-a6db-4d4d307cdf46:c0c11d0b-803a-4c83-b59e-25bfe891a71a,2cbcb46e-481b-4bc7-a6db-4d4d307cdf46:5d75458b-8850-4955-a6b7-97f70e3e39c8,2cbcb46e-481b-4bc7-a6db-4d4d307cdf46:fde2b510-acfb-4590-b39b-e8c4ff85770c,2cbcb46e-481b-4bc7-a6db-4d4d307cdf46:61098c7c-0a84-40cc-9a6f-ce0c7547283c,2cbcb46e-481b-4bc7-a6db-4d4d307cdf46:45ccaeb4-d739-4da1-9601-9d2e429fc840,2cbcb46e-481b-4bc7-a6db-4d4d307cdf46:d24be10d-6b0f-48a9-a0fc-9d85ff0c0ee7,2cbcb46e-481b-4bc7-a6db-4d4d307cdf46:2c63ab80-e1fe-4be6-9a40-17c7141dcb73,2cbcb46e-481b-4bc7-a6db-4d4d307cdf46:9848fa52-20e6-4716-a791-d371221e6ff1) |
| Collagen makes up 70-80% of the dry weight of the skin | x | [^24–38^](https://app.readcube.com/library/2cbcb46e-481b-4bc7-a6db-4d4d307cdf46/all?uuid=9644254536536102&item_ids=2cbcb46e-481b-4bc7-a6db-4d4d307cdf46:728d23d3-0922-447b-adc9-06c258ad7853,2cbcb46e-481b-4bc7-a6db-4d4d307cdf46:fdd2de07-1da1-437a-992c-29b78d2386c5,2cbcb46e-481b-4bc7-a6db-4d4d307cdf46:76c6dd2c-9629-46bf-a14a-a8771b8c486b,2cbcb46e-481b-4bc7-a6db-4d4d307cdf46:3fbfcf22-1235-42df-9f05-7c908515cd91,2cbcb46e-481b-4bc7-a6db-4d4d307cdf46:2c9132a9-768d-457d-9894-7f20ba43d53f,2cbcb46e-481b-4bc7-a6db-4d4d307cdf46:8c45a4b7-f440-4255-89e2-f1a9bdef1902,2cbcb46e-481b-4bc7-a6db-4d4d307cdf46:72db0a81-a4b8-4c84-bef8-2d99ae1fbb1a,2cbcb46e-481b-4bc7-a6db-4d4d307cdf46:1d3ca63a-d011-4d8c-a618-198a4c790f23,2cbcb46e-481b-4bc7-a6db-4d4d307cdf46:5597b9ff-920b-4f0d-abf9-3718ef703b56,2cbcb46e-481b-4bc7-a6db-4d4d307cdf46:8644ade1-6fc8-443d-874e-336be271c289,2cbcb46e-481b-4bc7-a6db-4d4d307cdf46:70848238-a07c-4008-b366-fea1bb5589ba,2cbcb46e-481b-4bc7-a6db-4d4d307cdf46:7c4842bd-f787-4257-aabe-729885c958d1,2cbcb46e-481b-4bc7-a6db-4d4d307cdf46:4aa7b8fe-5f1e-4ab0-ba69-aad9c03abc9f,2cbcb46e-481b-4bc7-a6db-4d4d307cdf46:7811ca70-7429-4912-b9ce-166cfc41ed6f,2cbcb46e-481b-4bc7-a6db-4d4d307cdf46:dce6061e-8176-4329-84a7-5400fd6544be) |
| Collagen 80% bones | x | [^39,40^](https://app.readcube.com/library/2cbcb46e-481b-4bc7-a6db-4d4d307cdf46/all?uuid=8325080005400644&item_ids=2cbcb46e-481b-4bc7-a6db-4d4d307cdf46:e5dffd6e-9936-4dc0-9c95-c63eceeb61c6,2cbcb46e-481b-4bc7-a6db-4d4d307cdf46:b7964200-0fa2-417a-9681-f16c37e10d18) |
| Collagen 90% bones | x | [^41–44^](https://app.readcube.com/library/2cbcb46e-481b-4bc7-a6db-4d4d307cdf46/all?uuid=8309256530898219&item_ids=2cbcb46e-481b-4bc7-a6db-4d4d307cdf46:f4350d82-b7d7-413e-b011-6affcd8199e9,2cbcb46e-481b-4bc7-a6db-4d4d307cdf46:54a0dded-2384-4b1e-888f-f02009f6aab6,2cbcb46e-481b-4bc7-a6db-4d4d307cdf46:1e20dbfa-6642-4c1c-84ce-a7bc8412c06a,2cbcb46e-481b-4bc7-a6db-4d4d307cdf46:27a89af4-ba88-4f7f-9348-ea8a6d816090) |
| Collagen 50-80% cartilage dry weight | x | [^45–52^](https://app.readcube.com/library/2cbcb46e-481b-4bc7-a6db-4d4d307cdf46/all?uuid=1174913878995405&item_ids=2cbcb46e-481b-4bc7-a6db-4d4d307cdf46:9721ac1e-4533-41ec-99f6-114cbab1e71d,2cbcb46e-481b-4bc7-a6db-4d4d307cdf46:c45fd423-689f-48fe-b5a7-a5b8ac31917f,2cbcb46e-481b-4bc7-a6db-4d4d307cdf46:fb036ca6-6cfd-4039-b0d9-ec39abac6db1,2cbcb46e-481b-4bc7-a6db-4d4d307cdf46:ee8efa19-6b95-4d8f-8a59-6577a61fb9f5,2cbcb46e-481b-4bc7-a6db-4d4d307cdf46:3f5d0bb5-1121-4763-811a-eef9dd1e8f5b,2cbcb46e-481b-4bc7-a6db-4d4d307cdf46:b02cf94a-9eba-4769-a468-0943c961157d,2cbcb46e-481b-4bc7-a6db-4d4d307cdf46:21bd8200-5973-4068-ac10-74115b96b523,2cbcb46e-481b-4bc7-a6db-4d4d307cdf46:3c91de9b-82e7-4be4-b5d3-dc717ae29c30) |

References:

[1. Hausman, R. E. Ocular extracellular matrices in development. *Progress in retinal and eye research* **26**, 162–188 (2007).
2. Gentle, A., Liu, Y., Martin, J. E., Conti, G. L. & McBrien, N. A. Collagen gene expression and the altered accumulation of scleral collagen during the development of high myopia. *The Journal of biological chemistry* **278**, 16587–16594 (2003).
3. Huang, W. *et al.* Collagen: a potential factor involved in the pathogenesis of glaucoma. *Medical science monitor basic research* **19**, 237–240 (2013).
4. Rada, J. A. S., Shelton, S. & Norton, T. T. The sclera and myopia. *Experimental eye research* **82**, 185–200 (2006).
5. McBrien, N. A., Cornell, L. M. & Gentle, A. Structural and ultrastructural changes to the sclera in a mammalian model of high myopia. *Invest Ophth Vis Sci* **42**, 2179–87 (2001).
6. Markov, P. P. et al. Bulk changes in posterior scleral collagen microstructure in human high myopia. *Mol Vis* **24**, 818–833 (2018).
7. Hatami-Marbini, H. & Pachenari, M. Hydration related changes in tensile response of posterior porcine sclera. *J Mech Behav Biomed* **104**, 10**35**62 (2020).
8. Zhao, F. et al. Cause and Effect Relationship between Changes in Scleral Matrix Metallopeptidase-2 Expression and Myopia Development in Mice. *Am J Pathology* **188**, 1754–1767 (2018).
9. Yang, Q. et al. A Potential Research Target for Scleral Remodeling: Effect of MiR-29a on Scleral Fibroblasts. *Ophthalmic Res* (2022) doi:10.1159/000525189.
10. Li, H. et al. BMP-2 Is Involved in Scleral Remodeling in Myopia Development. *Plos One* **10**, e0125219 (2015).
11. Kjær, M. Role of extracellular matrix in adaptation of tendon and skeletal muscle to mechanical loading. *Physiological reviews* **84**, 649–698 (2004).
12. Thorpe, C. T., Birch, H. L., Clegg, P. D. & Screen, H. R. C. The role of the non-collagenous matrix in tendon function. *International journal of experimental pathology* **94**, 248–259 (2013).
13. Screen, H. R. C., Berk, D. E., Kadler, K. E., Ramirez, F. & Young, M. F. Tendon functional extracellular matrix. *Journal of orthopaedic research : official publication of the Orthopaedic Research Society* **33**, 793–799 (2015).
14. Reuvers, J. et al. The mechanical properties of tail tendon fascicles from lubricin knockout, wild type and heterozygous mice. *Journal of Structural Biology* **176**, 41–45 (2011).
15. Yamamoto, E., Hayashi, K. & Yamamoto, N. Mechanical properties of collagen fascicles from stress-shielded patellar tendons in the rabbit. *Clinical biomechanics (Bristol, Avon)* **14**, 418–425 (1999).
16. D’souza, Z. et al. Collagen – structure, function and distribution in orodental tissues. *Journal of Global Oral Health* **2**, 134–139 (2020).
17. Taye, N., Karoulias, S. Z. & Hubmacher, D. The “other” 15–40%: The Role of Non‐Collagenous Extracellular Matrix Proteins and Minor Collagens in Tendon. *J Orthop Res* **38**, 23–35 (2020).
18. Noriega-González, D. C. et al. Effect of Vitamin C on Tendinopathy Recovery: A Scoping Review. *Nutrients* **14**, 2663 (20**22**).
19. Jerban, S. et al. Age-related decrease in collagen proton fraction in tibial tendons estimated by magnetization transfer modeling of ultrashort echo time magnetic resonance imaging (UTE-MRI). *Sci Rep-uk* **9**, 17974 (2019).
20. Svensson, R. B., Heinemeier, K. M., Couppé, C., Kjaer, M. & Magnusson, S. P. Effect of aging and exercise on the tendon. *J Appl Physiol* **121**, 1353–1362 (2017).
21. Picaut, L. et al. Pure dense collagen threads from extrusion to fibrillogenesis stability. *Biomed Phys Eng Express* **4**, 035008 (2018).
22. Frizziero, A. et al. Effect of training and sudden detraining on the patellar tendon and its enthesis in rats. *Bmc Musculoskelet Di* **12**, 20–20 (2011).
23. Mienaltowski, M. J. & Birk, D. E. Progress in Heritable Soft Connective Tissue Diseases. *Adv Exp Med Biol* **802**, 5–29 (2013).
**24**. Oikarinen, A. Aging of the skin connective tissue: how to measure the biochemical and mechanical properties of aging dermis. *Photodermatology, photoimmunology & photomedicine* **10**, 47–52 (1994).
25. Haapasaari, K. M. et al. Systemic therapy with estrogen or estrogen with progestin has no effect on skin collagen in postmenopausal women. *Maturitas* **27**, 153–162 (1997).
26. Jin, G., Prabhakaran, M. P. & Ramakrishna, S. Stem cell differentiation to epidermal lineages on electrospun nanofibrous substrates for skin tissue engineering. *Acta biomaterialia* **7**, 3113–3122 (2011).
27. George, J., Onodera, J. & Miyata, T. Biodegradable honeycomb collagen scaffold for dermal tissue engineering. *Journal of biomedical materials research. Part A* **87**, 1103–1111 (2008).
28. Luebberding, S., Krueger, N. & Kerscher, M. Mechanical properties of human skin in vivo: a comparative evaluation in 300 men and women. *Skin research and technology : official journal of International Society for Bioengineering and the Skin (ISBS) [and] International Society for Digital Imaging of Skin (ISDIS) [and] International Society for Skin Imaging (ISSI)* **20**, **12**7–135 (2014).
29. Tzaphlidou, M. The role of collagen and elastin in aged skin: an image processing approach. 35, 173–177 (2004).
30. Uitto, J. Biochemistry of the elastic fibers in normal connective tissues and its alterations in diseases. *Journal of Investigative Dermatology* **72**, 1–10 (1979).
31. Miyachi, Y. & Ishikawa, O. Dermal connective tissue metabolism in photoageing. *The Australasian journal of dermatology* **39**, 19–23 (1998).
32. Edwards, J. V. & Howley, P. S. Human neutrophil elastase and collagenase sequestration with phosphorylated cotton wound dressings. Journal of biomedical materials research. Part A **83**, 446–454 (2007).
33. Seehra, G. P. & Silver, F. H. Viscoelastic properties of acid- and alkaline-treated human dermis: a correlation between total surface charge and elastic modulus. Skin research and technology : official journal of International Society for Bioengineering and the Skin (ISBS) [and] International Society for Digital Imaging of Skin (ISDIS) [and] International Society for Skin Imaging (ISSI) 12, 190–198 (2006).
34. Bilaç, C., Şahin, M. T. & Öztürkcan, S. Chronic actinic damage of facial skin. *Clinics in dermatology* **32**, 752–762 (2014).
35. Kim, M. K. et al. Anti-wrinkle effects of Seungma-Galgeun-Tang as evidenced by the inhibition of matrix metalloproteinase-I production and the promotion of type-1 procollagen synthesis. *BMC complementary and alternative medicine* **16**, 116 (2016).
36. Tobin, D. J. Biochemistry of human skin—our brain on the outside. *Chem Soc Rev* **35**, 52–67 (2005).
37. Brett, D. ﻿A Review of Collagen and Collagen-based Wound Dressings. *Wounds Compend Clin Res Pract* **20**, 347–56 (2008).
38. Qin, Z., Robichaud, P., Quan, T. & USA, D. of D., University of Michigan Medical School, Ann Arbor, Michigan,. Oxidative stress and CCN1 protein in human skin connective tissue aging. *Aims Mol Sci* **3**, 269–279 (2016).
39. Daneault, A., Prawitt, J., Soulé, V. F., Coxam, V. & Wittrant, Y. Biological effect of hydrolyzed collagen on bone metabolism. *Critical reviews in food science and nutrition* **57**, 1922–1937 (2017).
40. Viguet-Carrin, S., Garnero, P. & Delmas, P. D. The role of collagen in bone strength. *Osteoporosis international : a journal established as result of cooperation between the European Foundation for Osteoporosis and the National Osteoporosis Foundation of the USA* **17**, 319–336 (2006).
41. Eriksen, E. F. et al. Serum markers of type I collagen formation and degradation in metabolic bone disease: correlation with bone histomorphometry. *Journal of bone and mineral research : the official journal of the American Society for Bone and Mineral Research* **8**, 127–132 (1993).
42. Fernández-Tresguerres-Hernández-Gil, I., Alobera-Gracia, M. A., del-Canto-Pingarrón, M. & Blanco-Jerez, L. Physiological bases of bone regeneration I. Histology and physiology of bone tissue. *Medicina oral, patologia oral y cirugia bucal* **11**, E47-51 (2006).
43. Tzaphlidou, M. Bone Architecture: Collagen Structure and Calcium/Phosphorus Maps. *J Biol Phys* **34**, 39–49 (2008).
44. Feng, X. Chemical and Biochemical Basis of Cell-Bone Matrix Interaction in Health and Disease. *Curr Chem Biology* **3**, 189–196 (2009).
45. Mow, V. C., Ratcliffe, A. & Poole, A. R. Cartilage and diarthrodial joints as paradigms for hierarchical materials and structures. *Biomaterials* **13**, 67–97 (1992).
46. Torzilli, P. A., Rose, D. E. & Dethmers, D. A. Equilibrium water partition in articular cartilage. *Biorheology* **19**, 519–537 (1982).
47. Darling, E. M., Hu, J. C. Y. & Athanasiou, K. A. Zonal and topographical differences in articular cartilage gene expression. Journal of orthopaedic research : official publication of the Orthopaedic Research Society 22, 1182–1187 (2004).
48. Fetter, N. L., Leddy, H. A., Guilak, F. & Nunley, J. A. Composition and transport properties of human ankle and knee cartilage. Journal of orthopaedic research : official publication of the Orthopaedic Research Society 24, 211–219 (2006).
49. USA, U. of W., Dept of Orthopaedics and Sports Medicine, Box 356500, Seattle, WA 98195-6500, Eyre, D., Weis, M. & Wu, J.-J. Articular cartilage collagen: an irreplaceable framework? *European Cells Mater* **22**, 57–63 (2006).
50. Fox, A. J. S., Bedi, A. & Rodeo, S. A. The basic science of articular cartilage: structure, composition, and function. *Sports health* **1**, 461–468 (2009).
51. Luo, Y. et al. The minor collagens in articular cartilage. *Protein Cell* **8**, 560–572 (2017).
52. Eyre, D. Collagen of articular cartilage. *Arthritis Res* **4**, 30–5 (2001).](https://app.readcube.com/library/?style=Scientific%20Reports+%7B%22language%22:%22en-US%22%7D)
