# Supplementary material for: Collagen constitutes about 12% in females and 17% in males of the total protein in mice
Source: Sci Rep. 2023 Mar 18;13:4490. doi: 10.1038/s41598-023-31566-z (PMC10024738; doi:10.1038/s41598-023-31566-z)
Supplement: Supplementary file 5 — Supplementary Table 5. [file 41598_2023_31566_MOESM5_ESM.docx]

Supplementary Table 5. Collagen content across species and tissues

| **Species** | **Strain** | **Breed** | **Sex** | **Age**  **[weeks]** | **# of animals** | **Whole Organism** | **Muscle** | **Skin** | **Cartilage, Tendon, ligaments** | **Liver** | **Spleen** | **Lung** | **Kidney** | **Heart** | **Additional tissues** | **Method** | **Sample preparation** | **Notes** | **Reference** |
| --- | --- | --- | --- | --- | --- | --- | --- | --- | --- | --- | --- | --- | --- | --- | --- | --- | --- | --- | --- |
| *Mus Musculus* | Albino | NS | male | NS | 10 | 1000+40 mg total collagen per 35+1 g of body weight |  |  |  |  |  |  |  |  |  | Measured hydroxyproline for collagen levels. Weighted animals. | Whole body, excluding kidneys and genital organs. | Collagen content estimated by 7.46 multiplied by hydroxyproline levels | ^[1](https://app.readcube.com/library/2cbcb46e-481b-4bc7-a6db-4d4d307cdf46/all?uuid=6012913369909664&item_ids=2cbcb46e-481b-4bc7-a6db-4d4d307cdf46:e81af430-20d5-4840-952c-cd8b08a522b5)^ |
| *Mus Musculus* | CF1 | inbred | male | 14 | 6 |  |  | 20+1.8 μg collagen to μg total protein  =20% |  |  |  | 10+1.1 μg collagen to μg total protein  =10% | 6+0.1 μg collagen to μg total protein  =6% |  | Oesophagus 18+1.1, Jejunum 7+1.1, Ileum 10+1.1, Colon 12+1.1, μg collagen to μg total protein | Measured hydroxyproline for collagen levels. BSA for total protein. | Paraffin-embedded and sectioned material. | Standard curve with gelatin (denatured collagen) for hydroxyproline measurements. | [^2^](https://app.readcube.com/library/2cbcb46e-481b-4bc7-a6db-4d4d307cdf46/all?uuid=8855434327171723&item_ids=2cbcb46e-481b-4bc7-a6db-4d4d307cdf46:c89de513-db7c-4d59-be00-815c38a8d48d) |
| *Rattus norvegicus domesticus* | Wistar albino | outbred | male | NS | 4 |  |  |  |  | 5.8+1 ug collagen/ mg protein =0.6% | 17.4+3 ug collagen/ mg protein =2% | 65+14 ug collagen/ mg protein =7% | 18.9+8ug collagen/ mg protein =2% | 17.0+4 ug collagen/ mg protein =2% |  | Binding of Sirius red F3BA to collagen and Fast green FCF to non-collagenous proteins. Confirmed with hydroxyproline and micro-Kieldahl procedure. One value shown for both methods, since similar results. | Formalin-fixed paraffin-embedded tissue sections. | Assumption one alpha chain of ca 96kDa contains about 100 4-hydroxyproline and proteins contain 16% nitrogen. | [^3^](https://app.readcube.com/library/2cbcb46e-481b-4bc7-a6db-4d4d307cdf46/all?uuid=8829755388375303&item_ids=2cbcb46e-481b-4bc7-a6db-4d4d307cdf46:d8017e73-a4c3-4bc4-a01f-362dbf5c1ab1) |
| *Mus Musculus* | Swiss albino | outbred | male and female | NS | 4 males & 4 females |  |  |  |  | 0.14+0.02 collagen nitrogen vs 3.25+0.06 total nitrogen =4% |  | 0.38+0.02 collagen nitrogen vs 2.23+0.04 total nitrogen =17% |  |  |  | Collagen extraction by sodium hydroxide. Insoluble fraction vs soluble fraction for nitrogen levels. Hydroxyproline for collagen. | Frozen tissue |  | [^4^](https://app.readcube.com/library/2cbcb46e-481b-4bc7-a6db-4d4d307cdf46/all?uuid=7040513306823661&item_ids=2cbcb46e-481b-4bc7-a6db-4d4d307cdf46:3397de30-3ba7-46eb-8f6b-9df96d5b26ae) |
| *Mus Musculus* | Swiss albino | outbred | male | 8 | 5 |  | 5.79+0.82 ug collagen /mg of gastrocnemius muscle tissue weight |  |  | 0.73+0.11 ug collagen /mg tissue weight |  | 4.64+0.67 ug collagen /mg tissue weight | 2.20+0.19 ug collagen /mg tissue weight | 2.82+0.18 ug collagen /mg heart ventricle tissue weight | 9.64+1.04 ug collagen /mg of pinna weight; 1.42+0.34 ug collagen /mg of intestine weight | Hydroxyproline for collagen. Wet tissue weight. | fresh tissue | Assumption collagen contains 14% 4-hydroxyproline. Hydroxyproline to collagen conversion factor of 7.14. | [^5^](https://app.readcube.com/library/2cbcb46e-481b-4bc7-a6db-4d4d307cdf46/all?uuid=7856532756311886&item_ids=2cbcb46e-481b-4bc7-a6db-4d4d307cdf46:8c59f485-4234-40bc-b08a-52cd2d93eab1) |
| *Rattus norvegicus domesticus* | Lewis | Inbred | male | 12 | 5 |  |  | 130.9+17.6 mg collagen /g tissue weight |  |  |  | 21.27+1.88 mg collagen /g tissue weight |  | 5.89+0.26 mg collagen /g tissue weight |  | Hydroxyproline for collagen. Wet tissue weight. | Frozen tissue |  | [^6^](https://app.readcube.com/library/2cbcb46e-481b-4bc7-a6db-4d4d307cdf46/all?uuid=08092157266697964&item_ids=2cbcb46e-481b-4bc7-a6db-4d4d307cdf46:c4677ec5-f7b5-4996-8068-af84adcbd2a0) |
| *Rattus norvegicus domesticus* | Albino | outbred | female | NS | 6 |  |  |  |  | 11.8+0.5 mg collagen / 7.8+0.3 g liver = 1.5 ug collagen /mg tissue weight |  |  |  |  |  | Hydroxyproline for collagen. Wet tissue weight. | fresh tissue | Hydroxyproline to collagen conversion factor of 7.46 | [^7^](https://app.readcube.com/library/2cbcb46e-481b-4bc7-a6db-4d4d307cdf46/all?uuid=9603070582911559&item_ids=2cbcb46e-481b-4bc7-a6db-4d4d307cdf46:a8404216-d269-49cb-8f6a-3b443dec623f) |
| Oryctolagus cuniculus | New Zealand white rabbit | outbred | male | 28 | 6 |  |  |  | 867.2+8.9 mg collagen /g of patellar tendon dry weight; 868.2+10.2 mg collagen /g of achilles tendon dry weight; 797.1+11.1 mg collagen /g of medial collateral ligament dry weight; 802.6+9.8 mg collagen /g of cruciate ligament dry weight |  |  |  |  |  |  | Hydroxyproline for collagen. | Dry tissue | Conversion hydroxyproline to collagen content not described. | [^8^](https://app.readcube.com/library/2cbcb46e-481b-4bc7-a6db-4d4d307cdf46/all?uuid=9549974861090662&item_ids=2cbcb46e-481b-4bc7-a6db-4d4d307cdf46:c3bef999-3869-4967-aab2-a9d416f59363) |
| Bos taurus | NS | NS | male | 72 | 2 |  | 2.24-5.59 % dry muscle weight. Percent range for different 18 muscles |  |  |  |  |  |  |  |  | Hydroxyproline for collagen | Fat-free dry tissue | “Two 18 month old steers were slaughtered…” Hydroxyproline to collagen conversion factor of 7.14 | [^9^](https://app.readcube.com/library/2cbcb46e-481b-4bc7-a6db-4d4d307cdf46/all?uuid=11214454442757327&item_ids=2cbcb46e-481b-4bc7-a6db-4d4d307cdf46:2635125a-46a1-42a2-a300-f451d9eb01ed) |

Legend:

Data is represented as mean + standard error.

NS = not specified

Discuss these issue in intro:

Not used, since report ug collagen per tissue (no weight indicated): [^10,11^](https://app.readcube.com/library/2cbcb46e-481b-4bc7-a6db-4d4d307cdf46/all?uuid=6851064824991167&item_ids=2cbcb46e-481b-4bc7-a6db-4d4d307cdf46:bf10be65-e3e3-4290-bf7a-8999f1698ef6,2cbcb46e-481b-4bc7-a6db-4d4d307cdf46:90a944a5-7c7c-4552-aea5-5062c00bcfe0)

Not used, although gm/100gm dry weight tissue, old method (not reliable) measures soluble collagen [^12^](https://app.readcube.com/library/2cbcb46e-481b-4bc7-a6db-4d4d307cdf46/all?uuid=4450495754520578&item_ids=2cbcb46e-481b-4bc7-a6db-4d4d307cdf46:af26df6a-8511-483d-8f8c-6bf5da954451)

Not used, since report ug hydroxyproline (not collagen converted): [^13^](https://app.readcube.com/library/2cbcb46e-481b-4bc7-a6db-4d4d307cdf46/all?uuid=10403391784334248&item_ids=2cbcb46e-481b-4bc7-a6db-4d4d307cdf46:c6defb2f-012a-4435-813b-bfa5665b1897)

Not used, since discarded insoluble fraction: [^14,15^](https://app.readcube.com/library/2cbcb46e-481b-4bc7-a6db-4d4d307cdf46/all?uuid=6407650563127244&item_ids=2cbcb46e-481b-4bc7-a6db-4d4d307cdf46:271d9bf7-6d65-4fb4-ab19-a82586dbb944,2cbcb46e-481b-4bc7-a6db-4d4d307cdf46:25a19374-506d-474a-ba66-22276e14334a)

References:

[1. HARKNESS, M. L., HARKNESS, R. D. & JAMES, D. W. The effect of a protein-free diet on the collagen content of mice. *The Journal of physiology* **144**, 307–313 (1958).
2. Schwartz, D. E., Choi, Y., Sandell, L. J. & Hanson, W. R. Quantitative analysis of collagen, protein and DNA in fixed, paraffin-embedded and sectioned tissue. *The Histochemical journal* **17**, 655–663 (1985).
3. León, A. L.-D. & Rojkind, M. A simple micromethod for collagen and total protein determination in formalin-fixed paraffin-embedded sections. *The journal of histochemistry and cytochemistry : official journal of the Histochemistry Society* **33**, 737–743 (1985).
4. Rosenkrantz, H., Esber, H. J. & Sprague, R. Lung hydroxyproline levels in mice exposed to cigarette smoke. *Life sciences* **8**, 571–576 (1969).
5. Patiyal, S. N. & Katoch, S. S. Tissue specific and variable collagen proliferation in Swiss albino mice treated with clenbuterol. *Physiological research* **55**, 97–103 (2006).
6. Mays, P. K., Bishop, J. E. & Laurent, G. J. Age-related changes in the proportion of types I and III collagen. *Mechanisms of ageing and development* **45**, 203–212 (1988).
7. HARKNESS, M. L. & HARKNESS, R. D. The collagen content of the liver in pregnancy and lactation. The Journal of physiology **134**, 135–138 (1956).
8. Amiel, D., Frank, C., Harwood, F., Fronek, J. & Akeson, W. Tendons and ligaments: a morphological and biochemical comparison. *Journal of orthopaedic research : official publication of the Orthopaedic Research Society* **1**, 257–265 (1984).
9. Dransfield, E. Intramuscular composition and texture of beef muscles. *Journal of the science of food and agriculture* **28**, 833–842 (1977).
10. Kliment, C. R., Englert, J. M., Crum, L. P. & Oury, T. D. A novel method for accurate collagen and biochemical assessment of pulmonary tissue utilizing one animal. *International journal of clinical and experimental pathology* **4**, 349–355 (2011).
11. Markova, M. S. *et al.* A role for the androgen receptor in collagen content of the skin. *Journal of Investigative Dermatology* **123**, 1052–1056 (2004).
12. Lowry, O. H., Gilligan, D. R. & Katersky, E. M. The determination of collagen and elastin in tissues with results obtained in various normal tissues from different species. *The Journal of biological chemistry* **139**, 795–804 (1941).
13. Takubo, Y. et al. Age-associated changes in elastin and collagen content and the proportion of types I and III collagen in the lungs of mice. *Experimental gerontology* **34**, 353–364 (1999).
14. Kimata, K., Barrach, H. J., Brown, K. S. & Pennypacker, J. P. Absence of proteoglycan core protein in cartilage from the cmd/cmd (cartilage matrix deficiency) mouse. The Journal of biological chemistry **256**, 6961–6968 (1981).
15. Bendall, J. R. The elastin content of various muscles of beef animals. Journal of the science of food and agriculture **18**, 553–558 (1967).](https://app.readcube.com/library/?style=Scientific%20Reports+%7B%22language%22:%22en-US%22%7D)
