# Supplementary material for: Collagen constitutes about 12% in females and 17% in males of the total protein in mice
Source: Sci Rep. 2023 Mar 18;13:4490. doi: 10.1038/s41598-023-31566-z (PMC10024738; doi:10.1038/s41598-023-31566-z)
Supplement: Supplementary file 6 — Supplementary Table 6. [file 41598_2023_31566_MOESM6_ESM.docx]

Supplementary Table 6. Collagen content of mammals

| **Statement: One-third or 30% of total protein is collagen in mammals** | **No citation or review cited** | **References** |
| --- | --- | --- |
| CONNECTIVE TISSUE takes part in the structure of most organs. Its main protein is collagen, which may thus be 30-40% of all the proteins of the body | x | [^1^](https://app.readcube.com/library/2cbcb46e-481b-4bc7-a6db-4d4d307cdf46/all?uuid=19597459587118393&item_ids=2cbcb46e-481b-4bc7-a6db-4d4d307cdf46:5abb789c-347c-42ef-b297-79580f33d4bb) |
| COLLAGENcomprises about 30°10of total body protein and undergoes very marked changes in properties during aging. | x | [^2^](https://app.readcube.com/library/2cbcb46e-481b-4bc7-a6db-4d4d307cdf46/all?uuid=6470841584569099&item_ids=2cbcb46e-481b-4bc7-a6db-4d4d307cdf46:c75e147d-98d1-4384-828b-22f9fa658bb1) |
| Collagen is the major supportive component of connective tissue and constitutes about 30% of the total body protein in mammals. | x | [^3^](https://app.readcube.com/library/2cbcb46e-481b-4bc7-a6db-4d4d307cdf46/all?uuid=5637498800545331&item_ids=2cbcb46e-481b-4bc7-a6db-4d4d307cdf46:9390fbe8-1356-4bff-8f2b-0786b6c95419) |
| Collagen is the most abundant protein (by weight) in animals, accounting for 30% of all proteins in mammals. | x | [^4^](https://app.readcube.com/library/2cbcb46e-481b-4bc7-a6db-4d4d307cdf46/all?uuid=7222155409089035&item_ids=2cbcb46e-481b-4bc7-a6db-4d4d307cdf46:c27a8468-f694-40df-af20-0a2f701bfbe9) |
| If one considers that protein comprises roughly 20% of body mass (8) and that 30% or more of total protein is represented by collagen (9, 10), and if it is assumed that type I collagen comprises at least 90% of total collagen, it can be calculated that an adult of 70 kg may contain more than 1 􏰅 1021 type I collagen monomers. | x | [^5^](https://app.readcube.com/library/2cbcb46e-481b-4bc7-a6db-4d4d307cdf46/all?uuid=2203467250077682&item_ids=2cbcb46e-481b-4bc7-a6db-4d4d307cdf46:112d122f-d061-4d1b-b028-d51e5948da28) |
| Collagen is an abundant structural protein in all animals. In humans, collagen comprises one- third of the total protein, accounts for three- quarters of the dry weight of skin, and is the most prevalent component of the extracellu- larmatrix(ECM). | x | [^6^](https://app.readcube.com/library/2cbcb46e-481b-4bc7-a6db-4d4d307cdf46/all?uuid=8710497511899369&item_ids=2cbcb46e-481b-4bc7-a6db-4d4d307cdf46:4cba8b20-c460-44e2-97dd-359b584b30f1) |
| Collagen is the major constituent of connective tissue in animals and the most abundant protein in mammals, comprising about 25% to 35% of all protein. | x | [^7^](https://app.readcube.com/library/2cbcb46e-481b-4bc7-a6db-4d4d307cdf46/all?uuid=035669015697935125&item_ids=2cbcb46e-481b-4bc7-a6db-4d4d307cdf46:8da9194a-184d-4c7e-9c13-273194257d65) |
| Collagen is the most abundant fibrous protein within the interstitial ECM and constitutes up to 30% of the total protein mass of a multicellular animal. | x | [^8^](https://app.readcube.com/library/2cbcb46e-481b-4bc7-a6db-4d4d307cdf46/all?uuid=032571158923254684&item_ids=2cbcb46e-481b-4bc7-a6db-4d4d307cdf46:c57a4d31-31b8-4d41-b5fc-8d5f9dc175f0) |
| Collagens are the most abundant proteins in mammals (30% of total protein mass). | x | [^9^](https://app.readcube.com/library/2cbcb46e-481b-4bc7-a6db-4d4d307cdf46/all?uuid=28074405760491505&item_ids=2cbcb46e-481b-4bc7-a6db-4d4d307cdf46:03a4490b-8c88-4af1-b407-9e3c6a100a7f) |
| They constitute one-third of AA in the collagen proteins which comprise approximately 30% of body proteins. | x | [^10^](https://app.readcube.com/library/2cbcb46e-481b-4bc7-a6db-4d4d307cdf46/all?uuid=6331621983732298&item_ids=2cbcb46e-481b-4bc7-a6db-4d4d307cdf46:61bdf87d-d87e-4cfe-8d72-cda6895c8aae) |
| Collagen is the most abundant (~ 30%) protein in the body, and the largest component of the ECM, where it serves an essential structural role as provider of tensile strength to tissues and organs. | x | [^11^](https://app.readcube.com/library/2cbcb46e-481b-4bc7-a6db-4d4d307cdf46/all?uuid=762870258051845&item_ids=2cbcb46e-481b-4bc7-a6db-4d4d307cdf46:703a3b18-d975-44e9-a6c4-fde1b0614765) |
| Collagens constitute nearly 30% of all proteins in our body. | x | [^12^](https://app.readcube.com/library/2cbcb46e-481b-4bc7-a6db-4d4d307cdf46/all?uuid=363172200485266&item_ids=2cbcb46e-481b-4bc7-a6db-4d4d307cdf46:8af8a8e8-379e-4efb-9d75-ded3e3e4fc5e) |
| Collagen comprises 25-30% of the protein content of the whole body especially in mammals (Muller & Werner, 2003). | x | [^13^](https://app.readcube.com/library/2cbcb46e-481b-4bc7-a6db-4d4d307cdf46/all?uuid=4812054766425211&item_ids=2cbcb46e-481b-4bc7-a6db-4d4d307cdf46:ae643e5f-37a6-47ba-82cd-4117859e08a7) |
| Collagen is the most abundant and ubiquitous protein in animal origin, which comprising approximately 30% of total protein. | x | [^14^](https://app.readcube.com/library/2cbcb46e-481b-4bc7-a6db-4d4d307cdf46/all?uuid=3154250389655868&item_ids=2cbcb46e-481b-4bc7-a6db-4d4d307cdf46:dfa6d357-8ab6-43e6-aa7c-8667bb048467) |
| Collagens constitute approximately 30% of all proteins in the body, with type I collagen as the most ubiquitous collagen (Muiznieks and Keeley, 2013). | x | [^15^](https://app.readcube.com/library/2cbcb46e-481b-4bc7-a6db-4d4d307cdf46/all?uuid=44873398494440975&item_ids=2cbcb46e-481b-4bc7-a6db-4d4d307cdf46:1a9b41c7-a72e-4c90-a09a-926ecb836a54) |
| Collagen makes up to 30% of the mass of vertebrates and lays their structural framework. Due to its ubiquity and importance, collagen has been nicknamed the “steel of biological materials”. | x | [^16^](https://app.readcube.com/library/2cbcb46e-481b-4bc7-a6db-4d4d307cdf46/all?uuid=792164192743093&item_ids=2cbcb46e-481b-4bc7-a6db-4d4d307cdf46:e30c8dca-3b4a-4c8c-8a1f-5bb67e6c7d0c) |
| Collagen is a major class of structural proteins in bone, skin, cartilage and connective tissue (Liu et al., 2007; Ogawa et al., 2003; Bateman et al., 1996). It plays an important role in tissue development and is the most abundant protein in vertebrates, constituting about 30% of the total. | x | [^17^](https://app.readcube.com/library/2cbcb46e-481b-4bc7-a6db-4d4d307cdf46/all?uuid=3593362879222357&item_ids=2cbcb46e-481b-4bc7-a6db-4d4d307cdf46:b22c6984-8ae4-408f-b73f-bbeb4cb0e100) |
| Collagen is estimated that in the case of mammals, it is 25–30% of the dry weight [10]. | x | [^18^](https://app.readcube.com/library/2cbcb46e-481b-4bc7-a6db-4d4d307cdf46/all?uuid=11220438886544248&item_ids=2cbcb46e-481b-4bc7-a6db-4d4d307cdf46:c2a652da-ac76-446b-af66-622b04f42d68) |
| Collagen comprises over 30% of the protein mass of the human body and is an abundant structural protein in all animals (1). | x | [^19^](https://app.readcube.com/library/2cbcb46e-481b-4bc7-a6db-4d4d307cdf46/all?uuid=7728815656231105&item_ids=2cbcb46e-481b-4bc7-a6db-4d4d307cdf46:ff80058d-161b-4423-a534-dea428fcf7f3) |
| Collagen is a major component of fibrous connective tissues such as tendons and bone (12). It is the most abundant protein and accounts for 25–35% of total body protein content in mammals. | x | [^20^](https://app.readcube.com/library/2cbcb46e-481b-4bc7-a6db-4d4d307cdf46/all?uuid=36878573529419156&item_ids=2cbcb46e-481b-4bc7-a6db-4d4d307cdf46:07af0909-17fe-43e3-867c-2df58294346c) |
| Collagen is the most abundant protein (approximately 30% by weight of total protein) in human’s body which consists in extracellular matrix [31]. | x | [^21^](https://app.readcube.com/library/2cbcb46e-481b-4bc7-a6db-4d4d307cdf46/all?uuid=8163223020821756&item_ids=2cbcb46e-481b-4bc7-a6db-4d4d307cdf46:608a2554-6ecf-4d9d-ac14-6f346b7c9eb9) |
| Collagens represent 30% of the total protein mass in the body (Ricard-Blum, 2011) and are therefore the most abundant proteins in mammals. | x | [^22^](https://app.readcube.com/library/2cbcb46e-481b-4bc7-a6db-4d4d307cdf46/all?uuid=07287338137698074&item_ids=2cbcb46e-481b-4bc7-a6db-4d4d307cdf46:e5dffd6e-9936-4dc0-9c95-c63eceeb61c6) |
| In mammals, collagens constitute about 30% of the total protein mass in the body and are the major component of the ECM [2]. | x | [^23^](https://app.readcube.com/library/2cbcb46e-481b-4bc7-a6db-4d4d307cdf46/all?uuid=6567496661320089&item_ids=2cbcb46e-481b-4bc7-a6db-4d4d307cdf46:d1bd2e3d-3756-40a9-9a50-2719f118b606) |
| Collagen represents the chief structural protein account- ing for approximately 30% of all vertebrate body protein. | x | [^24^](https://app.readcube.com/library/2cbcb46e-481b-4bc7-a6db-4d4d307cdf46/all?uuid=2925830550396179&item_ids=2cbcb46e-481b-4bc7-a6db-4d4d307cdf46:388aa3e7-1382-4dff-a827-d5e90e40b50a) |
| They constitute one-third of AA in the collagen proteins which comprise approximately 30% of body proteins. | x | [^10^](https://app.readcube.com/library/2cbcb46e-481b-4bc7-a6db-4d4d307cdf46/all?uuid=005843196662335237&item_ids=2cbcb46e-481b-4bc7-a6db-4d4d307cdf46:61bdf87d-d87e-4cfe-8d72-cda6895c8aae) |
| Collagens are the most abundant proteins, accounting for about a third of all proteins in the body, while Hyp accounts for 10% to 15% of all its amino acid residues [1]. | x | [^25^](https://app.readcube.com/library/2cbcb46e-481b-4bc7-a6db-4d4d307cdf46/all?uuid=6329570480487242&item_ids=2cbcb46e-481b-4bc7-a6db-4d4d307cdf46:93510c43-f1e7-4f9e-aabb-ca978ee1d74d) |
| The abundance of Hyp among the residues in animal proteins is ∼4%, a value calculated from the abundance of collagen amongst animal proteins (1/3 ) and that of Hyp within collagen (∼38% × 1/3 ) (Ramshaw et al., 1998). | x | [^26^](https://app.readcube.com/library/2cbcb46e-481b-4bc7-a6db-4d4d307cdf46/all?uuid=0035803070569917494&item_ids=2cbcb46e-481b-4bc7-a6db-4d4d307cdf46:bbf6119c-c673-486a-bb54-c2706ecc5215) |
| Collagen constitutes one-third of dry body mass in vertebrates and is essential for the structure and function of a wide range of organs 53,54. | x | [^27^](https://app.readcube.com/library/2cbcb46e-481b-4bc7-a6db-4d4d307cdf46/all?uuid=9400691740583983&item_ids=2cbcb46e-481b-4bc7-a6db-4d4d307cdf46:b12dc26d-f589-453e-8246-57f3620c8735) |
| Collagen is the most abundant protein and represents one third of all proteins in humans. | x | [^28^](https://app.readcube.com/library/2cbcb46e-481b-4bc7-a6db-4d4d307cdf46/all?uuid=046271460126029784&item_ids=2cbcb46e-481b-4bc7-a6db-4d4d307cdf46:122f2612-f729-4179-b95c-9859b410f821) |
| Collagen is the most abundant protein in the human body, accounting for more than 30% of the total protein [1,2]. | x | [^29^](https://app.readcube.com/library/2cbcb46e-481b-4bc7-a6db-4d4d307cdf46/all?uuid=026674725035573554&item_ids=2cbcb46e-481b-4bc7-a6db-4d4d307cdf46:091010a6-5d2f-4155-9c03-9fca3d6683fc) |
| Collagens are the most abundant proteins in mammals accounting for approximately 30% of total protein mass [1]. | x | [^30^](https://app.readcube.com/library/2cbcb46e-481b-4bc7-a6db-4d4d307cdf46/all?uuid=929108196018817&item_ids=2cbcb46e-481b-4bc7-a6db-4d4d307cdf46:6b0b623e-bd5d-4a5d-aaee-47a09415446f) |
| Collagens consti- tute approximately 30% of all proteins in the body, with type I collagen as the most ubiquitous collagen (Muiznieks and Keeley, 2013). | x | [^15^](https://app.readcube.com/library/2cbcb46e-481b-4bc7-a6db-4d4d307cdf46/all?uuid=5174486623963263&item_ids=2cbcb46e-481b-4bc7-a6db-4d4d307cdf46:1a9b41c7-a72e-4c90-a09a-926ecb836a54) |
| Collagen is ubiquitous within various tissues, constituting approxi- mately 25%–30% of all body protein. | x | [^31^](https://app.readcube.com/library/2cbcb46e-481b-4bc7-a6db-4d4d307cdf46/all?uuid=8017905671328636&item_ids=2cbcb46e-481b-4bc7-a6db-4d4d307cdf46:0440bc32-107e-4b0f-ba91-905ad9d8741a) |
| Collagens represent 30% of total protein mass in mammals, providing a fundamental structural component of extracellular matrix (ECM) in all connective and interstitial tissue (Gelse et al., 2003). | x | [^32^](https://app.readcube.com/library/2cbcb46e-481b-4bc7-a6db-4d4d307cdf46/all?uuid=2606659958181594&item_ids=2cbcb46e-481b-4bc7-a6db-4d4d307cdf46:949fb65c-f28e-45e6-b094-83b0e7b56218) |
| Collagen is the most abundant protein found in the mammalian body, making up approximately 30% of the total body protein. | x | [^33^](https://app.readcube.com/library/2cbcb46e-481b-4bc7-a6db-4d4d307cdf46/all?uuid=11507260445039735&item_ids=2cbcb46e-481b-4bc7-a6db-4d4d307cdf46:7feecd99-f821-478d-ba72-d60b5aa3e06a) |
| Collagens are major structural proteins in the extracellular matrix, making up about one-third of protein mass in higher animals. | x | [^34^](https://app.readcube.com/library/2cbcb46e-481b-4bc7-a6db-4d4d307cdf46/all?uuid=33210564240903295&item_ids=2cbcb46e-481b-4bc7-a6db-4d4d307cdf46:19a937ad-54e0-4171-a813-cbc3f9350bb2) |
| Collagens form up to 30% of all proteins in the human body and serve principally as supportive and protective scaffolds; however, they also make up the key proteins for a range of vital processes at work within the organism.1 | x | [^35^](https://app.readcube.com/library/2cbcb46e-481b-4bc7-a6db-4d4d307cdf46/all?uuid=03461290561262376&item_ids=2cbcb46e-481b-4bc7-a6db-4d4d307cdf46:0fde06bf-4992-4202-9734-07783165ad6b) |
| Collagen comprises the main structural protein in vertebrates, accounting for about 30 % of total protein, and is found in the connective tissues of animals (i.e., skin, scale, bone, tendon, etc.) (Foegeding et al., 1996). | x | [^36^](https://app.readcube.com/library/2cbcb46e-481b-4bc7-a6db-4d4d307cdf46/all?uuid=06968800669051656&item_ids=2cbcb46e-481b-4bc7-a6db-4d4d307cdf46:ac5e101e-858b-4aed-84cb-3ebafe468d79) |
|  |  |  |
| **Statement: 25% of total protein is collagen in mammals** |  |  |
| Collagen is the major protein of the extracellular matrix (ECM) and is the most abundant protein found in mammals, comprising 25% of the total protein and 70% to 80% of skin (dry weight). | x | [^37^](https://app.readcube.com/library/2cbcb46e-481b-4bc7-a6db-4d4d307cdf46/all?uuid=8591967968771224&item_ids=2cbcb46e-481b-4bc7-a6db-4d4d307cdf46:7811ca70-7429-4912-b9ce-166cfc41ed6f) |
| Collagen: it is the major components of the extracellular matrix and the most abundant protein in mammals, making up to 􏰉25% of the total protein to support and maintain cell and tissue structures. | x | [^38^](https://app.readcube.com/library/2cbcb46e-481b-4bc7-a6db-4d4d307cdf46/all?uuid=31118525852749035&item_ids=2cbcb46e-481b-4bc7-a6db-4d4d307cdf46:a6d719fd-a25c-4d19-a195-17452716a2ff) |
| Collagen constitutes 25% of total proteins in vertebrates. | x | [^39^](https://app.readcube.com/library/2cbcb46e-481b-4bc7-a6db-4d4d307cdf46/all?uuid=23227593409766722&item_ids=2cbcb46e-481b-4bc7-a6db-4d4d307cdf46:5077f61c-a1c1-4c48-94eb-740c15e08315) |
| Collagens are the most abundant class of ECM proteins in the human body comprising 25% of total protein mass, collagen I being a major interstitial matrix protein and collagen IV being a major basement mem- brane protein (for a detailed review, see Ref. [45]). | x | [^40^](https://app.readcube.com/library/2cbcb46e-481b-4bc7-a6db-4d4d307cdf46/all?uuid=028107454983490876&item_ids=2cbcb46e-481b-4bc7-a6db-4d4d307cdf46:a4f9d8ec-0eb0-4093-92aa-6fedcd885f35) |
| Collagen is also the most abundant protein in mammals, a major component of connective tissue, accounting for around 25% of total protein content. | x | [^41^](https://app.readcube.com/library/2cbcb46e-481b-4bc7-a6db-4d4d307cdf46/all?uuid=9385196157967681&item_ids=2cbcb46e-481b-4bc7-a6db-4d4d307cdf46:071c1e2c-2333-4ffb-a0ce-09d8c60cee15) |
|  |  |  |
| **Statement: Collagen is the most abundant protein** |  |  |
| Collagen is the major protein of the extracellular matrix (ECM) and is the most abundant protein found in mammals, comprising 25% of the total protein and 70% to 80% of skin (dry weight). | x | [^37^](https://app.readcube.com/library/2cbcb46e-481b-4bc7-a6db-4d4d307cdf46/all?uuid=609613791151317&item_ids=2cbcb46e-481b-4bc7-a6db-4d4d307cdf46:7811ca70-7429-4912-b9ce-166cfc41ed6f) |
| Collagen is the most abundant protein (by weight) in animals, accounting for 30% of all proteins in mammals. | x | [^4^](https://app.readcube.com/library/2cbcb46e-481b-4bc7-a6db-4d4d307cdf46/all?uuid=361443150512758&item_ids=2cbcb46e-481b-4bc7-a6db-4d4d307cdf46:c27a8468-f694-40df-af20-0a2f701bfbe9) |
| Collagen: it is the major components of the extracellular matrix and the most abundant protein in mammals, making up to 􏰉25% of the total protein to support and maintain cell and tissue structures. | x | [^38^](https://app.readcube.com/library/2cbcb46e-481b-4bc7-a6db-4d4d307cdf46/all?uuid=01047874772038615&item_ids=2cbcb46e-481b-4bc7-a6db-4d4d307cdf46:a6d719fd-a25c-4d19-a195-17452716a2ff) |
| Collagen is the most prominent protein of human tissues. | x | [^42^](https://app.readcube.com/library/2cbcb46e-481b-4bc7-a6db-4d4d307cdf46/all?uuid=4908654601351138&item_ids=2cbcb46e-481b-4bc7-a6db-4d4d307cdf46:4887578c-866b-4328-a138-5408ccaf5db2) |
| Collagen is the major constituent of connective tissue in animals and the most abundant protein in mammals, comprising about 25% to 35% of all protein. | x | [^7^](https://app.readcube.com/library/2cbcb46e-481b-4bc7-a6db-4d4d307cdf46/all?uuid=7877848023782967&item_ids=2cbcb46e-481b-4bc7-a6db-4d4d307cdf46:8da9194a-184d-4c7e-9c13-273194257d65) |
| Collagen is the most abundant fibrous protein within the interstitial ECM and constitutes up to 30% of the total protein mass of a multicellular animal. | x | [^8^](https://app.readcube.com/library/2cbcb46e-481b-4bc7-a6db-4d4d307cdf46/all?uuid=7305243453466425&item_ids=2cbcb46e-481b-4bc7-a6db-4d4d307cdf46:c57a4d31-31b8-4d41-b5fc-8d5f9dc175f0) |
| Collagens are the most abundant proteins in mammals (30% of total protein mass). | x | [^9^](https://app.readcube.com/library/2cbcb46e-481b-4bc7-a6db-4d4d307cdf46/all?uuid=6512676900636137&item_ids=2cbcb46e-481b-4bc7-a6db-4d4d307cdf46:03a4490b-8c88-4af1-b407-9e3c6a100a7f) |
| Collagen is the most abundant (~ 30%) protein in the body, and the largest component of the ECM, where it serves an essential structural role as provider of tensile strength to tissues and organs. | x | [^11^](https://app.readcube.com/library/2cbcb46e-481b-4bc7-a6db-4d4d307cdf46/all?uuid=43324423239298815&item_ids=2cbcb46e-481b-4bc7-a6db-4d4d307cdf46:703a3b18-d975-44e9-a6c4-fde1b0614765) |
| Collagen is the most abundant and ubiquitous protein in animal origin, which comprising approximately 30% of total protein. | x | [^14^](https://app.readcube.com/library/2cbcb46e-481b-4bc7-a6db-4d4d307cdf46/all?uuid=5352697109376694&item_ids=2cbcb46e-481b-4bc7-a6db-4d4d307cdf46:dfa6d357-8ab6-43e6-aa7c-8667bb048467) |
| Collagen is a major class of structural proteins in bone, skin, cartilage and connective tissue (Liu et al., 2007; Ogawa et al., 2003; Bateman et al., 1996). It plays an important role in tissue development and is the most abundant protein in vertebrates, constituting about 30% of the total. | x | [^17^](https://app.readcube.com/library/2cbcb46e-481b-4bc7-a6db-4d4d307cdf46/all?uuid=8658360645983683&item_ids=2cbcb46e-481b-4bc7-a6db-4d4d307cdf46:b22c6984-8ae4-408f-b73f-bbeb4cb0e100) |
| Collagen comprises over 30% of the protein mass of the human body and is an abundant structural protein in all animals (1). | x | [^19^](https://app.readcube.com/library/2cbcb46e-481b-4bc7-a6db-4d4d307cdf46/all?uuid=9998522434589524&item_ids=2cbcb46e-481b-4bc7-a6db-4d4d307cdf46:ff80058d-161b-4423-a534-dea428fcf7f3) |
| Collagen is the most abundant protein (approximately 30% by weight of total protein) in human’s body which consists in extracellular matrix [31]. | x | [^21^](https://app.readcube.com/library/2cbcb46e-481b-4bc7-a6db-4d4d307cdf46/all?uuid=2262607859677056&item_ids=2cbcb46e-481b-4bc7-a6db-4d4d307cdf46:608a2554-6ecf-4d9d-ac14-6f346b7c9eb9) |
| Collagens represent 30% of the total protein mass in the body (Ricard-Blum, 2011) and are therefore the most abundant proteins in mammals. | x | [^22^](https://app.readcube.com/library/2cbcb46e-481b-4bc7-a6db-4d4d307cdf46/all?uuid=509191425836898&item_ids=2cbcb46e-481b-4bc7-a6db-4d4d307cdf46:e5dffd6e-9936-4dc0-9c95-c63eceeb61c6) |
| Collagens — the most abundant proteins in the body by weight — are the main structural proteins in the extracellular matrix (ECM) of various tissues, including cartilage, bone, blood vessels, skin, and other connective tissues (Figure 1). | x | [^43^](https://app.readcube.com/library/2cbcb46e-481b-4bc7-a6db-4d4d307cdf46/all?uuid=9649877685115623&item_ids=2cbcb46e-481b-4bc7-a6db-4d4d307cdf46:4f0a9408-e49a-4b36-9c23-c8809250fe89) |
| Collagen is the most ubiquitous protein found in animals (from sponges to humans). | x | [^44^](https://app.readcube.com/library/2cbcb46e-481b-4bc7-a6db-4d4d307cdf46/all?uuid=48197406767067574&item_ids=2cbcb46e-481b-4bc7-a6db-4d4d307cdf46:b5d7da35-e145-4cf3-bb07-9d0f06301c9b) |
| Collagen is the most abundant protein found in humans and other animals (Shoulders and Raines 2009; Pallela, Ehrlich, and Bhatnagar 2016). | x | [^45^](https://app.readcube.com/library/2cbcb46e-481b-4bc7-a6db-4d4d307cdf46/all?uuid=016783919855017038&item_ids=2cbcb46e-481b-4bc7-a6db-4d4d307cdf46:b5cfc360-9cc9-4ab9-8320-e1cc67d74335) |
| Collagens are the most abundant protein present in vertebrates and invertebrate organisms and play major roles in their structural organizations, body flexibility, and elastic properties. | x | [^46^](https://app.readcube.com/library/2cbcb46e-481b-4bc7-a6db-4d4d307cdf46/all?uuid=10550257885425651&item_ids=2cbcb46e-481b-4bc7-a6db-4d4d307cdf46:deea440e-0bb8-44fe-b4a5-c6fdae6d7a3e) |
| Collagen is among the most abundant protein in animals, where it plays a major structural role in the extracellular matrix, including in all vertebrates and invertebrates. It also plays critical roles in molecular and cellular interactions in the extracellular matrix, defining the shape and form of tis- sues. | x | [^47^](https://app.readcube.com/library/2cbcb46e-481b-4bc7-a6db-4d4d307cdf46/all?uuid=5617144224558249&item_ids=2cbcb46e-481b-4bc7-a6db-4d4d307cdf46:bfeb3532-06c2-4eaa-8f26-331ecd2a3700) |
| Collagens are the most abundant proteins, accounting for about a third of all proteins in the body, while Hyp accounts for 10% to 15% of all its amino acid residues [1]. | x | [^25^](https://app.readcube.com/library/2cbcb46e-481b-4bc7-a6db-4d4d307cdf46/all?uuid=5573895744220311&item_ids=2cbcb46e-481b-4bc7-a6db-4d4d307cdf46:93510c43-f1e7-4f9e-aabb-ca978ee1d74d) |
| Collagen is the most abundant protein in animals, and the major component of connective tissue (Shoulders and Raines, 2009) | x | [^26^](https://app.readcube.com/library/2cbcb46e-481b-4bc7-a6db-4d4d307cdf46/all?uuid=10857428006612624&item_ids=2cbcb46e-481b-4bc7-a6db-4d4d307cdf46:bbf6119c-c673-486a-bb54-c2706ecc5215) |
| Collagens, the most abundant proteins in mammals, are defined by their triple-helical structures and distinctive Gly-Xaa-Yaa repeating sequence, where Xaa is often proline and Yaa, hydroxyproline (Hyp/O). | x | [^48^](https://app.readcube.com/library/2cbcb46e-481b-4bc7-a6db-4d4d307cdf46/all?uuid=7626584795072966&item_ids=2cbcb46e-481b-4bc7-a6db-4d4d307cdf46:205e4d9e-2ee6-4c12-8193-bfa5544bb06f) |
| Collagen is the most abundant protein and represents one third of all proteins in humans….  Collagen is the most abundant protein in the human body and there- fore cells must use a large amount of energy for procollagen biosynthesis. | x | [^28^](https://app.readcube.com/library/2cbcb46e-481b-4bc7-a6db-4d4d307cdf46/all?uuid=6720006145280479&item_ids=2cbcb46e-481b-4bc7-a6db-4d4d307cdf46:122f2612-f729-4179-b95c-9859b410f821) |
| Collagen is the most abundant protein in the human body, accounting for more than 30% of the total protein [1,2]. | x | [^29^](https://app.readcube.com/library/2cbcb46e-481b-4bc7-a6db-4d4d307cdf46/all?uuid=34776182598148486&item_ids=2cbcb46e-481b-4bc7-a6db-4d4d307cdf46:091010a6-5d2f-4155-9c03-9fca3d6683fc) |
| Collagens are the most abundant proteins in mammals accounting for approximately 30% of total protein mass [1]. | x | [^30^](https://app.readcube.com/library/2cbcb46e-481b-4bc7-a6db-4d4d307cdf46/all?uuid=41928878395440405&item_ids=2cbcb46e-481b-4bc7-a6db-4d4d307cdf46:6b0b623e-bd5d-4a5d-aaee-47a09415446f) |
| Collagen is the most abundant protein in vertebrates. | x | [^49^](https://app.readcube.com/library/2cbcb46e-481b-4bc7-a6db-4d4d307cdf46/all?uuid=0013843833175524178&item_ids=2cbcb46e-481b-4bc7-a6db-4d4d307cdf46:6b071ab0-95d7-4227-b777-801a23835708) |
| Among many core matrisome proteins, collagen represents by far the most abundant protein that constitutes the bulk (90% dry weight) of skin [3]. | x | [^50^](https://app.readcube.com/library/2cbcb46e-481b-4bc7-a6db-4d4d307cdf46/all?uuid=7729945613058062&item_ids=2cbcb46e-481b-4bc7-a6db-4d4d307cdf46:dce6061e-8176-4329-84a7-5400fd6544be) |
| Collagen is the body’s most abundant protein. | x | [^51^](https://app.readcube.com/library/2cbcb46e-481b-4bc7-a6db-4d4d307cdf46/all?uuid=41654221966308114&item_ids=2cbcb46e-481b-4bc7-a6db-4d4d307cdf46:5d22fae8-8bf9-484b-90b6-d299444d27df) |
| Collagen is the most abundant protein found in the mammalian body, making up approximately 30% of the total body protein… Collagen is the most abundant structural protein found in the vertebrate body… | x | [^33^](https://app.readcube.com/library/2cbcb46e-481b-4bc7-a6db-4d4d307cdf46/all?uuid=6071313178923174&item_ids=2cbcb46e-481b-4bc7-a6db-4d4d307cdf46:7feecd99-f821-478d-ba72-d60b5aa3e06a) |
| Proline constitutes about 10% of total amino acids (AAs) in collagen, which accounts for one-third of proteins in mammals [24]. | x | [^52^](https://app.readcube.com/library/2cbcb46e-481b-4bc7-a6db-4d4d307cdf46/all?uuid=5395368445948392&item_ids=2cbcb46e-481b-4bc7-a6db-4d4d307cdf46:8ac91148-16fe-4993-a602-4ef15d73d835) |
| Collagen is also the most abundant protein in mammals, a major component of connective tissue, accounting for around 25% of total protein content. | x | [^41^](https://app.readcube.com/library/2cbcb46e-481b-4bc7-a6db-4d4d307cdf46/all?uuid=2696649486471673&item_ids=2cbcb46e-481b-4bc7-a6db-4d4d307cdf46:071c1e2c-2333-4ffb-a0ce-09d8c60cee15) |
| Collagen is the most abundant protein in animals… Collagen is the major extracellular component of connective tissues and is the most abundant protein in animals (1–3). | x | [^53^](https://app.readcube.com/library/2cbcb46e-481b-4bc7-a6db-4d4d307cdf46/all?uuid=9170837384963728&item_ids=2cbcb46e-481b-4bc7-a6db-4d4d307cdf46:eefaa16a-d4f5-4eaa-980f-94de3625ce37) |
| Collagens are the most abundant proteins in mammals, mainly in the extracellular matrix (ECM)1. | x | [^54^](https://app.readcube.com/library/2cbcb46e-481b-4bc7-a6db-4d4d307cdf46/all?uuid=9987206949750865&item_ids=2cbcb46e-481b-4bc7-a6db-4d4d307cdf46:733eea8e-9a43-4d51-b7ed-bcd11ff4f62f) |
| Collagen is the most abundant protein in vertebrates. | x | [^55^](https://app.readcube.com/library/2cbcb46e-481b-4bc7-a6db-4d4d307cdf46/all?uuid=3476845269330162&item_ids=2cbcb46e-481b-4bc7-a6db-4d4d307cdf46:b411a704-fba6-4ed4-a6e0-5fdcf5189a9a) |
| Collagen is the most abundant protein in the body and is the main component of the extracellular matrix (ECM) that scaffolds all somatic cells, tissues, and organs.11 | x | [^56^](https://app.readcube.com/library/2cbcb46e-481b-4bc7-a6db-4d4d307cdf46/all?uuid=17146742185906738&item_ids=2cbcb46e-481b-4bc7-a6db-4d4d307cdf46:79978f9e-c8d8-4920-96a0-e9197a52b1a6) |

Note: As the main component of connective tissue, it is the most abundant protein in mammals,[1] making up from 25% to 35% of the whole-body protein content. (<https://en.wikipedia.org/wiki/Collagen>, accessed 05.10.22)

There are papers that state both that collagen is “one-third or 30% of total protein” and “most abundant protein” (Figure below).


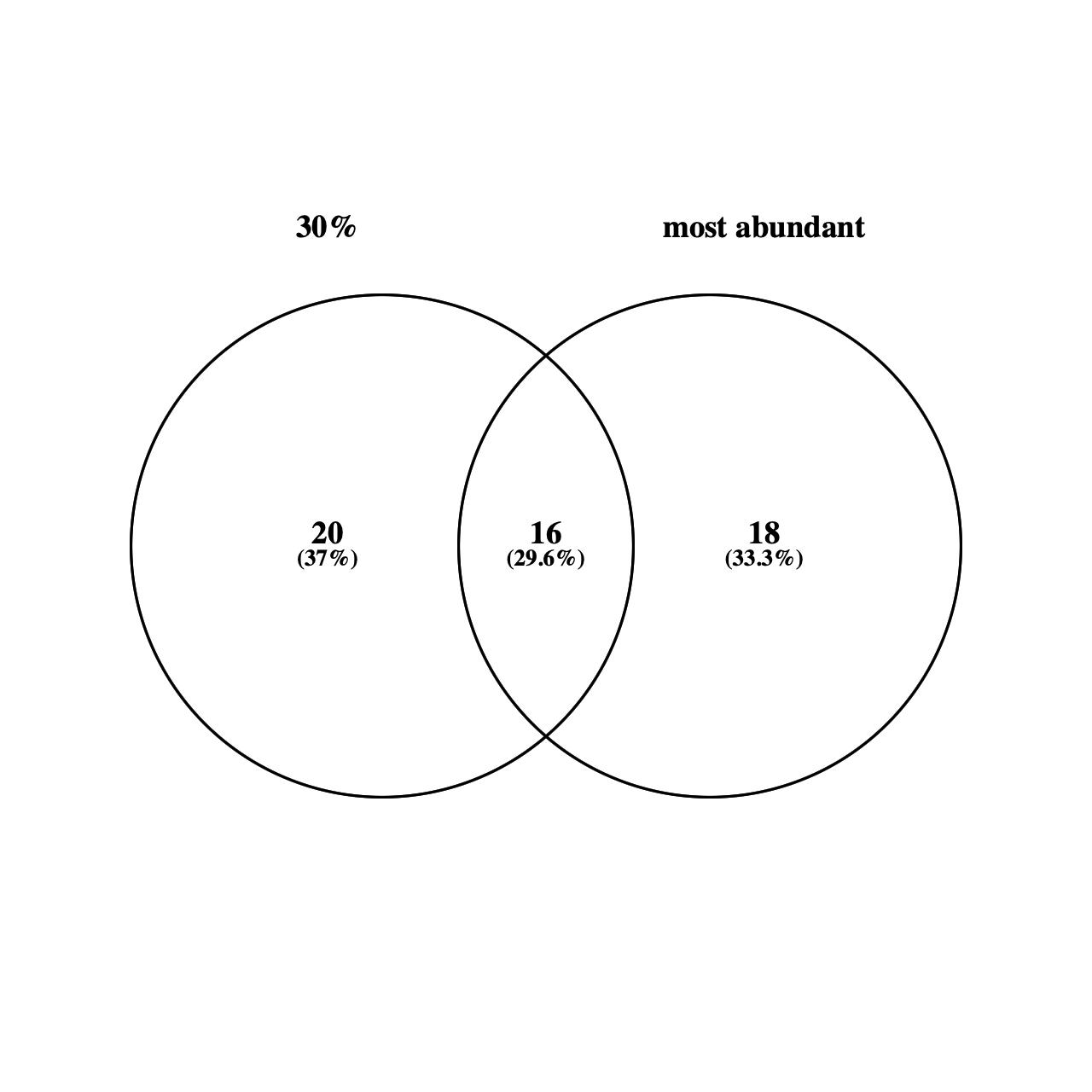


References

[1. Verzár, F. Aging of the collagen fiber. *International review of connective tissue research* **2**, 243–300 (1964).
2. Hamlin, C. R., Luschin, J. H. & Kohn, R. R. Aging of collagen: Comparative rates in four mammalian species. *Experimental gerontology* **15**, 393–398 (1980).
3. Wachem, P. B. van & Luyn, M. van. *Collagen Derived Materials*. vol. 28 (2001).
4. Patino, M. G., Neiders, M. E., Andreana, S., Noble, B. & Cohen, R. E. Collagen: An Overview. *Implant Dent* **11**, 280–285 (2002).
5. Lullo, G. A. D., Sweeney, S. M., Korkko, J., Ala-Kokko, L. & Antonio, J. D. S. Mapping the ligand-binding sites and disease-associated mutations on the most abundant protein in the human, type I collagen. *The Journal of biological chemistry* **277**, 4223–4231 (2002).
6. Shoulders, M. D. & Raines, R. T. Collagen structure and stability. *Annual review of biochemistry* **78**, 929–958 (2009).
7. Shimizu, K. *et al.* Molecular size of collagen peptide reverses the permeability of Caco-2 cells. *Bioscience, Biotechnology, and Biochemistry* **74**, 1123–1125 (2010).
8. Frantz, C., Stewart, K. M. & Weaver, V. M. The extracellular matrix at a glance. *Journal of Cell Science* **123**, 4195–4200 (2010).
9. Ricard-Blum, S. The collagen family. *Cold Spring Harbor perspectives in biology* **3**, a004978–a004978 (2011).
10. Wu, G. et al. Proline and hydroxyproline metabolism: implications for animal and human nutrition. *Amino Acids* **40**, 1053–1063 (2011).
11. Muiznieks, L. D. & Keeley, F. W. Molecular assembly and mechanical properties of the extracellular matrix: A fibrous protein perspective. *Biochimica et biophysica acta* **1832**, 866–875 (2013).
12. Pokidysheva, E. et al. Biological role of prolyl 3-hydroxylation in type IV collagen. *Proceedings of the National Academy of Sciences of the United States of America* **111**, 161–166 (2014).
13. Silvipriya, K. et al. Collagen: Animal Sources and Biomedical Application. *Journal of Applied Pharmaceutical Science* 123–127 (2015) doi:10.7324/japs.2015.50322.
14. Hashim, P, Baker & Hashim, M. Collagen in food and beverage industries. *IRFJ* **22**, 1–8 (2015).
15. Dragsbæk, K. et al. Matrix Metalloproteinase Mediated Type I Collagen Degradation — An Independent Risk Factor for Mortality in Women. *Ebiomedicine* **2**, 723–729 (2015).
16. Sherman, V. R., Yang, W. & Meyers, M. A. The materials science of collagen. *Journal of the mechanical behavior of biomedical materials* **52**, 22–50 (2015).
17. Jankangram, W., Chooluck, S. & Pomthong, B. Comparison of the properties of collagen extracted from dried jellyfish and dried squid. *African Journal of Biotechnology* **15**, 642–648 (2016).
18. Tylingo, R., Gorczyca, G., Mania, S., Szweda, P. & Milewski, S. Preparation and characterization of porous scaffolds from chitosan-collagen-gelatin composite. *REACT* **103**, 131–140 (2016).
19. Dittmore, A. et al. Internal strain drives spontaneous periodic buckling in collagen and regulates remodeling. Proceedings of the National Academy of Sciences **113**, 8436–8441 (2016).
20. Perła-Kajan, J. et al. N-Homocysteinylation impairs collagen cross-linking in cystathionine β-synthase-deficient mice: a novel mechanism of connective tissue abnormalities. *The FASEB Journal* **30**, 3810–3821 (2016).
21. Cheng, X. et al. Isolation, Characterization and Evaluation of Collagen from Jellyfish Rhopilema esculentum Kishinouye for Use in Hemostatic Applications. *PloS one* **12**, e0169731 (2017).
22. Daneault, A., Prawitt, J., Soulé, V. F., Coxam, V. & Wittrant, Y. Biological effect of hydrolyzed collagen on bone metabolism. *Critical reviews in food science and nutrition* **57**, 1922–1937 (2017).
23. Teuscher, A. C., Statzer, C., Pantasis, S., Bordoli, M. R. & Ewald, C. Y. Assessing Collagen Deposition During Aging in Mammalian Tissue and in Caenorhabditis elegans. *Methods Mol Biology Clifton N J* **1944**, 169–188 (2019).
24. Friess, W. Collagen--biomaterial for drug delivery. *European J Pharm Biopharm Official J Arbeitsgemeinschaft Fur Pharmazeutische Verfahrenstechnik E V* **45**, 113–36 (1998).
25. Belostotsky, R. & Frishberg, Y. Catabolism of Hydroxyproline in Vertebrates: Physiology, Evolution, Genetic Diseases and New siRNA Approach for Treatment. *Int J Mol Sci* **23**, 1005 (20**22**).
26. Gorres, K. L. & Raines, R. T. Prolyl 4-hydroxylase. *Crit Rev Biochem Mol* **45**, 106–124 (2010).
27. Unlu, G. et al. Phenome-based approach identifies RIC1-linked Mendelian syndrome through zebrafish models, biobank associations and clinical studies. *Nat Med* **26**, 98–109 (2020).
28. Ishikawa, Y. & Bächinger, H. P. A molecular ensemble in the rER for procollagen maturation. *Biochimica Et Biophysica Acta Bba - Mol Cell Res* **1833**, 2479–2491 (2013).
29. Kirkness, M. W., Lehmann, K. & Forde, N. R. Mechanics and structural stability of the collagen triple helix. *Curr Opin Chem Biol* **53**, 98–105 (2019).
30. Alcaide-Ruggiero, L., Molina-Hernández, V., Granados, M. M. & Domínguez, J. M. Main and Minor Types of Collagens in the Articular Cartilage: The Role of Collagens in Repair Tissue Evaluation in Chondral Defects. Int J Mol Sci 22, 13329 (2021).
31. Holwerda, A. M. & Loon, L. J. C. van. The impact of collagen protein ingestion on musculoskeletal connective tissue remodeling: a narrative review. *Nutr Rev* **80**, nuab083- (2021).
32. Paradiso, F. et al. Marine Collagen Substrates for 2D and 3D Ovarian Cancer Cell Systems. *Frontiers Bioeng Biotechnology* **7**, 343 (2019).
33. Noorzai, S. & Verbeek, C. J. R. Biotechnological Applications of Biomass. (2021) doi:**10**.5772/intechopen.94266.
34. Persikov, A. V. & Brodsky, B. Unstable molecules form stable tissues. *Proc National Acad Sci* **99**, 1101–1103 (2002).
35. Rýglová, Š., Braun, M. & Suchý, T. Collagen and Its Modifications—Crucial Aspects with Concern to Its Processing and Analysis. *Macromol Mater Eng* **302**, 1600460 (2017).
36. Moniruzzaman, S. Md. et al. Characterization of Acid- and Pepsin-soluble Collagens Extracted from Scales of Carp and Lizardfish Caught in Japan, Bangladesh and Vietnam with a Focus on Thermostability. *Food Sci Technol Res* **25**, 331–340 (2019).
37. Brett, D. ﻿A Review of Collagen and Collagen-based Wound Dressings. *Wounds Compend Clin Res Pract* **20**, 347–56 (2008).
38. Taguchi, T. & Razzaque, M. S. The collagen-specific molecular chaperone HSP47: is there a role in fibrosis? *Trends in molecular medicine* **13**, 45–53 (2007).
39. Muralidharan, N., Shakila, R. J., Sukumar, D. & Jeyasekaran, G. Skin, bone and muscle collagen extraction from the trash fish, leather jacket (Odonus niger) and their characterization. *Journal of food science and technology* **50**, 1106–1113 (2013).
40. Rafaeva, M. & Erler, J. T. Framing cancer progression: influence of the organ‐ and tumour‐specific matrisome. *Febs J* **287**, 1454–1477 (2020).
41. Shenoy, M. et al. Collagen Structure, Synthesis, and Its Applications: A Systematic Review. *Cureus* **14**, e24856 (2022).
42. Bayan, C., Levitt, J. M., Miller, E., Kaplan, D. & Georgakoudi, I. Fully automated, quantitative, noninvasive assessment of collagen fiber content and organization in thick collagen gels. *Journal of applied physics* **105**, 102042 (2009).
43. Bielajew, B. J., Hu, J. C. & Athanasiou, K. A. Collagen: quantification, biomechanics and role of minor subtypes in cartilage. *Nat Rev Mater* **5**, 730–747 (2020).
44. Oosterlaken, B. M., Vena, M. P. & With, G. In Vitro Mineralization of Collagen. *Adv Mater* **33**, 2004418 (2021).
45. Picker, J. et al. Prokaryotic Collagen-Like Proteins as Novel Biomaterials. Frontiers Bioeng Biotechnology 10, 840939 (2022).
46. Pallela, R., Ehrlich, H. & Bhatnagar, I. Marine Sponges: Chemicobiological and Biomedical Applications. 373–381 (2016) doi:10.1007/978-81-322-2794-6_20.
47. Ramshaw, J. A. M. Biomedical applications of collagens. *J Biomed Mater Res Part B Appl Biomaterials* **104**, 665–675 (2016).
48. Chow, W. Y. et al. Hydroxyproline Ring Pucker Causes Frustration of Helix Parameters in the Collagen Triple Helix. *Sci Rep-uk* **5**, 12556 (2015).
49. Michelacci, Y. M. Collagens and proteoglycans of the corneal extracellular matrix. *Braz J Med Biol Res* **36**, 1037–1046 (2003).
50. Qin, Z., Robichaud, P., Quan, T. & USA, D. of D., University of Michigan Medical School, Ann Arbor, Michigan,. Oxidative stress and CCN1 protein in human skin connective tissue aging. *Aims Mol Sci* **3**, 269–279 (2016).
51. Tobin, D. J. Introduction to skin aging. *J Tissue Viability* **26**, 37–46 (2017).
52. Karna, E., Szoka, L., Huynh, T. Y. L. & Palka, J. A. Proline-dependent regulation of collagen metabolism. *Cell Mol Life Sci* **77**, 1911–1918 (2020).
53. Zhang, Z. et al. Broadly conserved roles of TMEM131 family proteins in intracellular collagen assembly and secretory cargo trafficking. *Sci Adv* **6**, eaay7667 (2020).
54. Wang, Y. et al. Structure of Vibrio collagenase VhaC provides insight into the mechanism of bacterial collagenolysis. *Nat Commun* **13**, 566 (2022).
55. Pati, F., Dhara, S. & Adhikari, B. Fish Collagen: A Potential Material for Biomedical Application. *2010 Ieee Students Technology Symposium Techsym* 34–38 (2010) doi:10.1109/techsym.2010.5469184.
56. Upreti, T., Wolfe, K. M., Bavel, N. V., Anikovskiy, M. & Labouta, H. I. Collagen – a newly discovered major player in protein corona formation on nanoparticles. *Phys Chem Chem Phys* **24**, 5610–5617 (2022).](https://app.readcube.com/library/?style=Scientific%20Reports+%7B%22language%22:%22en-US%22%7D)
