# Supplementary material for: Collagen constitutes about 12% in females and 17% in males of the total protein in mice
Source: Sci Rep. 2023 Mar 18;13:4490. doi: 10.1038/s41598-023-31566-z (PMC10024738; doi:10.1038/s41598-023-31566-z)
Supplement: Supplementary file 12 — Supplementary Figures. [file 41598_2023_31566_MOESM12_ESM.pdf]

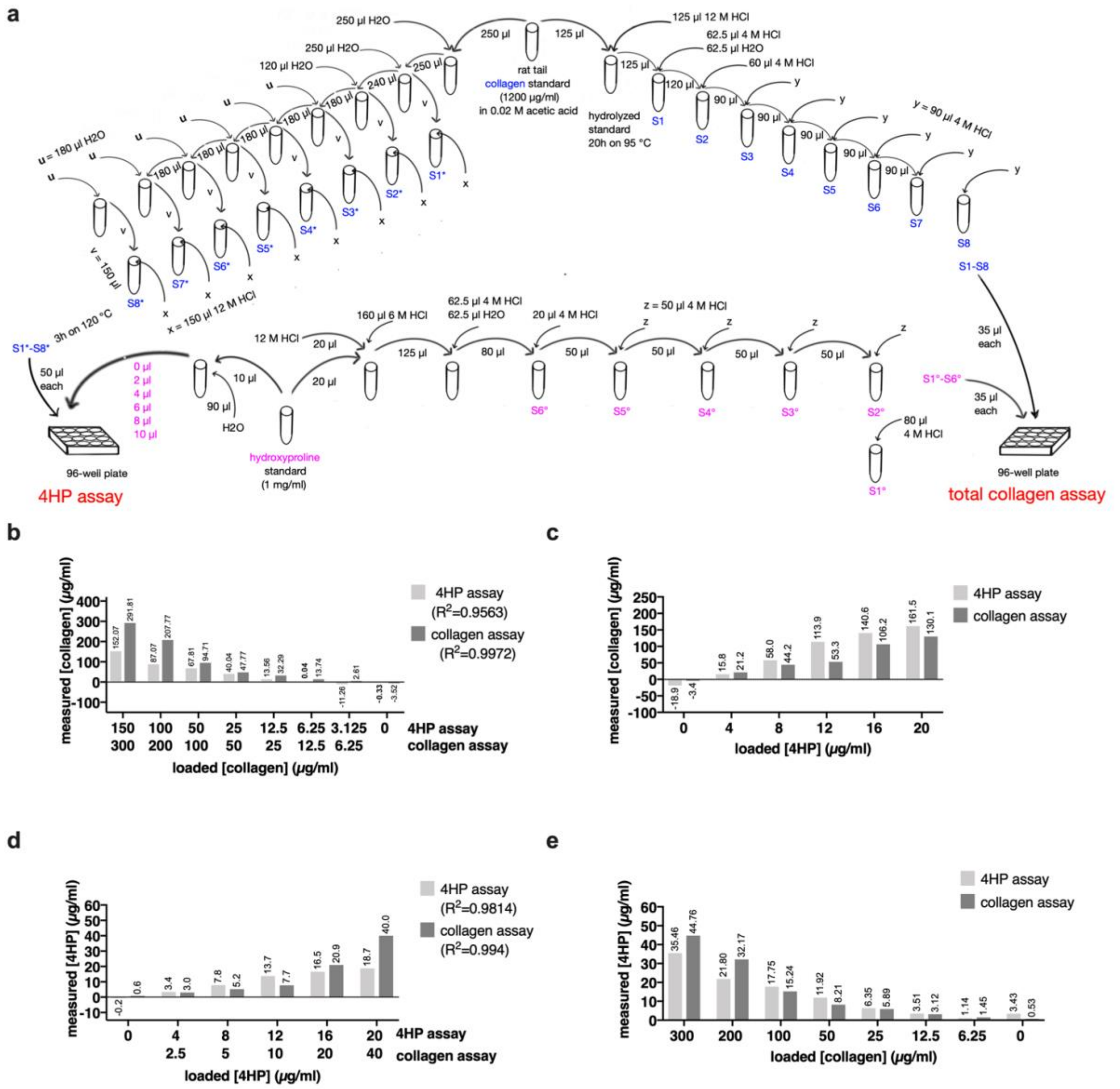

Supplementary Fig. 1. Collagen validation standard curves of the 4HP and collagen assay

a, schematic representation of the 4HP and total collagen assay standard curve preparation. The collagen assay standard (rat tail collagen, 1200µg/mL) was prepared according to the protocol for the collagen assay (right). For the 4HP assay (left), 250µL of the collagen standard was diluted with 250µL water (600µg/mL). This solution was diluted 1:2 with water (H2O) (300µg/mL). 240µL of the new dilution was mixed with 120µL water (200µg/mL). A 180µL aliquot was transferred to a new tube and diluted with 180µL H2O (u) (100µg/mL). This 1:2 dilution was repeated 4 times (50µg/mL, 25µg/mL, 12.5µg/mL, 6.25µg/mL). S1* is the blank. To be in the range of the 4HP standard curve, 150µL (v) of each sample was pipetted into a new tube (S1*-S8*) and diluted with 150µL 12M HCl (x). After hydrolyzation (3h on 120°C), 50µL of each sample was pipetted on a 96-well plate. The hydroxyproline standard (1mg/mL) was prepared according to the protocol for the 4HP assay (left). The 4HP assay concentrations need to be divided by 0.05 to convert the concentrations from µg/well into µg/mL (since 50µL of each S* were loaded). To be able to generate a 4HP standard curve on the collagen assay

plate (right), the standard was diluted 1:10 with HCl (0.1mg/mL in 6M HCl). 125µL of this concentration was mixed with 62.5µL 4M HCl and 62.5µL water. 80µL of this dilution was transferred and mixed with 20µL 4M HCl (S6°= 40µg/mL). The further standard concentrations were reached by consecutive 1:2 dilutions. (S5°= 20µg/mL, S4°= 10µg/mL, S3°= 5µg/mL, S2°= 2.5µg/mL). S1° is the blank. With the concentrations mentioned, collagen and a 4HP standard curve were generated for each assay. **b** and **d,** depict the measured collagen (b) and 4HP (d) values (µg/mL), which were used to generate the standard curve and correspond to the loaded, known concentrations. The top row on the x-axis indicates the concentrations (µg/mL) loaded in the 4HP assay, and the bottom row shows the ones loaded in the collagen assay. R^{2}'s represents the coefficient of determination of the resulting standard curves. **c,** compares the measured collagen values (µg/mL) of the two assays correlating with the loaded 4HP concentrations (µg/mL). For this, the collagen standard curve was considered on the two plates. Since different 4HP concentrations were loaded on the two assays, the rule of proportion was used to make the assays comparable. **e**, compares the measured 4HP values obtained from the loaded collagen concentrations. This time the 4HP standard curve was used on both plates.

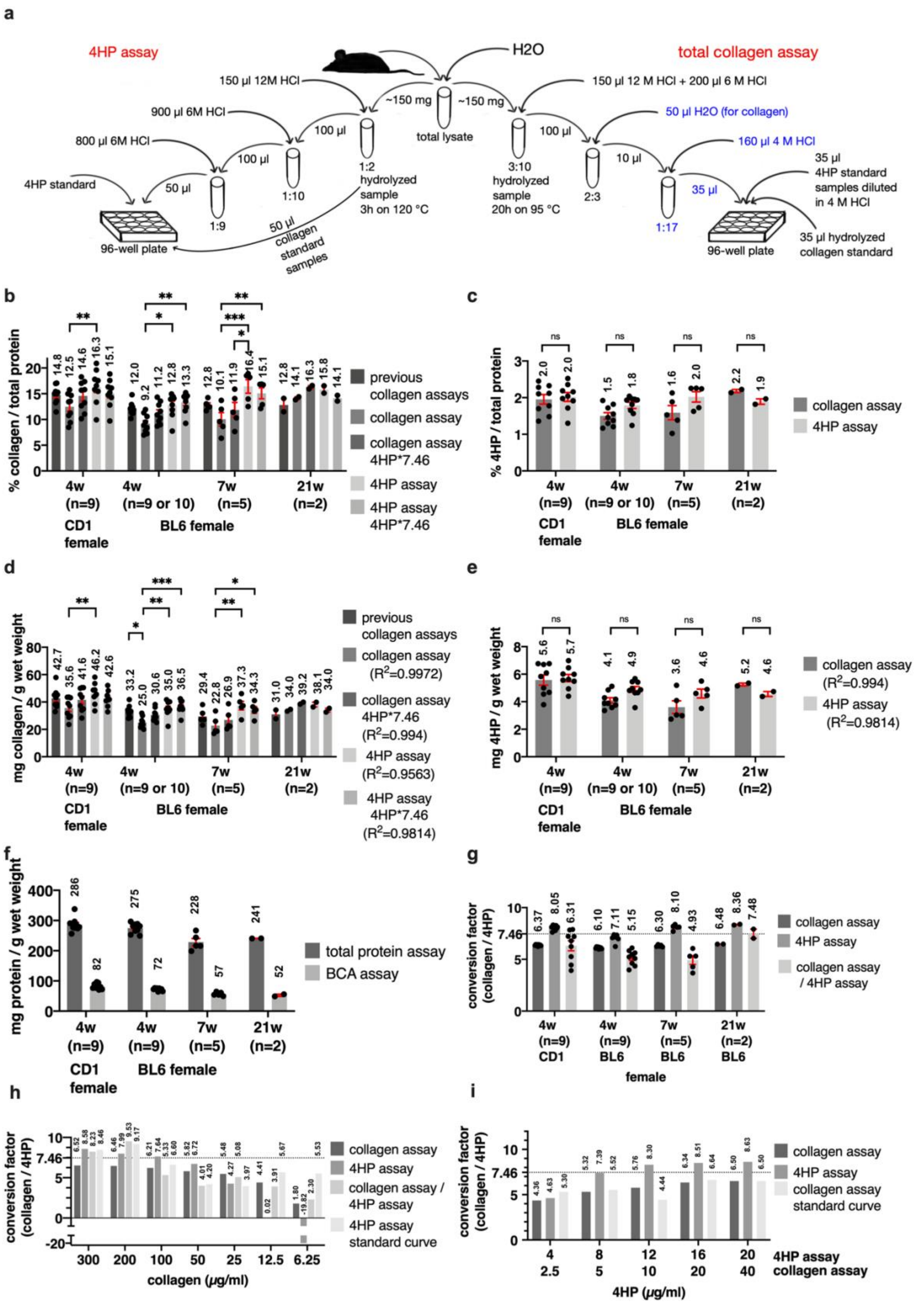

Supplementary Fig. 2. Comparison of the 4HP and collagen assay

a, schematic representation of the 4HP and total collagen assay procedure. The mice were diluted with water and aliquoted. An equal amount of the total lysate was used for the 4HP assay and for the collagen assay. For the total collagen assay, the lysates were diluted 3:10 to a final 6M HCl concentration and hydrolyzed for 20h at 95°C. This hydrolysate was diluted 2:3 with water and 1:17 with 4M HCl. 35 µL of the normal hydrolyzed collagen standard, 35 µL of 4HP standard samples derived from the 4HP assay standard solution, and 35 µL of the samples were loaded on a 96-well plate. For the 4HP assay, the lysates were diluted 1:2 with 12M HCl and hydrolyzed for 3h at 120°C. The hydrolysate was further diluted (1:10 and 1:9) with 6M HCl. 50 µL of these samples and 50 µL of the collagen standard dilutions, which were not further diluted after hydrolyzation, were loaded on a 96-well plate next to the normal 4HP standard.

b and **c,** show a percentage of collagen (b) or 4HP (c) to protein comparison of the different assays. The mean of the 3 previous protein assays was used for normalization. **d** and **e**, compare values (mg/g) of collagen or 4HP, respectively. **b** and **d**, 7.46 is the 4HP to collagen conversion factor cited in the literature (Neuman and Logan, 1950). The 4HP concentration of the corresponding assay is multiplied by this factor to get the collagen concentration. **f**, depicts the protein levels (mg/g) from the total protein assay and the BCA assay. **g**, illustrates the means ± SEM of the empirical conversion factors of the animal groups mentioned on the x-axis. The factor is derived by dividing the collagen concentration of a sample by its 4HP concentration. Three different methods were used to calculate it. Either the values used for the calculation were taken from the same plate (collagen assay, 4HP assay) or from different plates (collagen assay / 4HP assay).

b-g, the first group consists of 4 weeks old CD1 females. The others are C57BL/6 females. The numbers indicate the age in weeks (w). Shown is the mean ± SEM of 2-10 biological replicates. (values of biological replicates are derived from 1-3 technical replicates), 2way ANOVA posthoc Tukey (a,c) or Sidak (b,d).

h and **i** show the conversion factors calculated by using the collagen and 4HP standard curves, respectively. Four or three different methods were used to calculate it. Either the values used for the calculation were taken from the same plate (collagen assay, 4HP assay), from different plates (collagen assay / 4HP assay), or the calculation was done by using the measured values and the concentrations indicated in the x-axis (4HP assay standard curve or collagen assay standard curve, respectively). See Supplementary Table 9 for details.

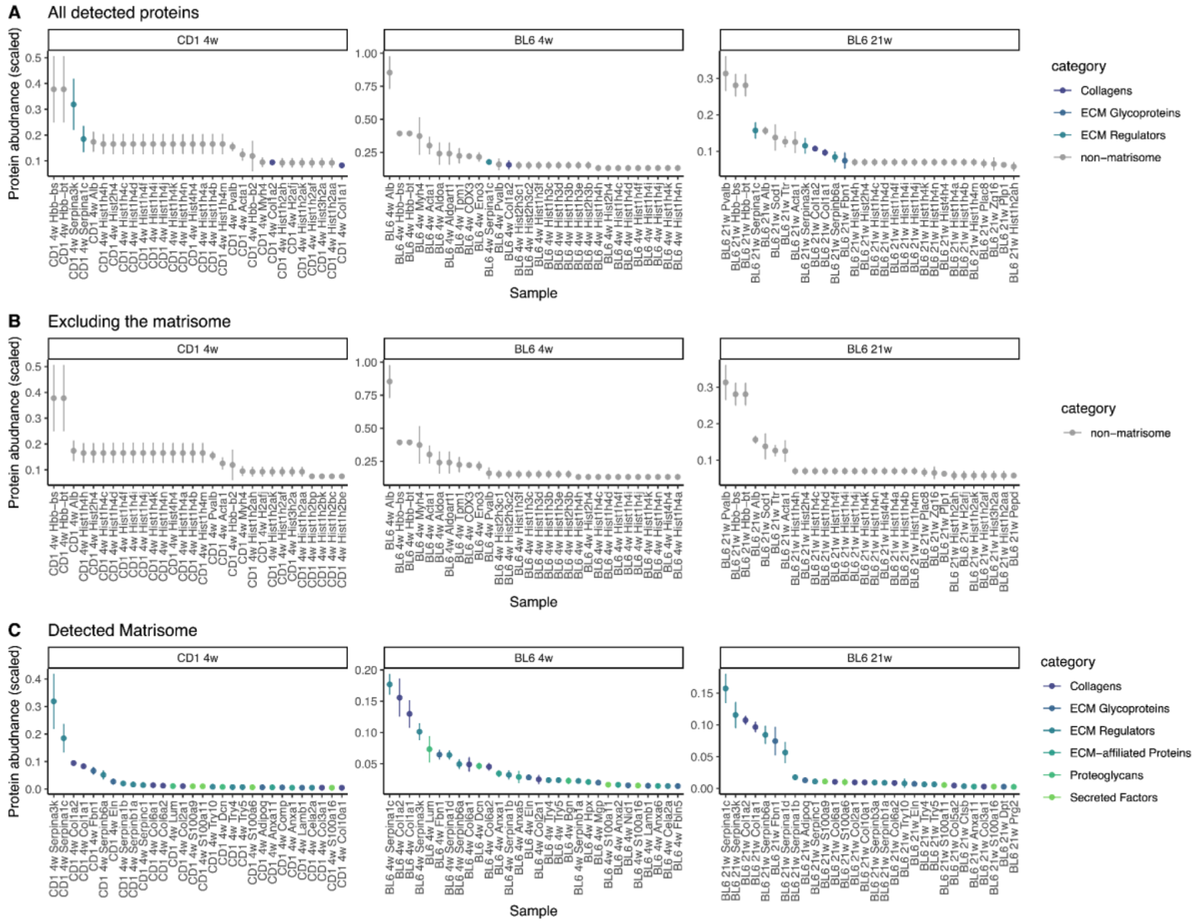

Supplementary Fig. 3. Top 30 most abundant proteins of wild-type mice

The scaled protein abundances are displayed in horizontal panels for each mouse cohort (CD1 4 weeks, BL6 4 weeks, BL6 21 weeks) for all detected proteins (a), all non-matrisome proteins (b), and exclusively the matrisome (c). The matrisome membership is represented by the color of each data point indicating its matrisome category. Details are in Supplementary Table 11.
